# Chlorhexidine for umbilical cord care: game-changer for newborn survival?

**DOI:** 10.9745/GHSP-D-12-00014

**Published:** 2013-03-21

**Authors:** Steve Hodgins, YV Pradhan, Leela Khanal, Shyam Upreti, Naresh Pratap KC

**Affiliations:** a Formerly with MCHIP-Maternal and Child Health Integrated Program and now on the Saving Newborn Lives project; b Formerly with the Ministry of Health and Population, Government of Nepal; c John Snow, Inc./Nepal; d Ministry of Health and Population, Government of Nepal

## Abstract

A simple technology with potential to prevent 500,000 global neonatal deaths annually.

## NEWBORN MORTALITY: AN INTRACTABLE PROBLEM?

Newborn mortality has been a persistent challenge, with reductions in neonatal deaths lagging behind declines in post-neonatal child mortality in most low-income countries.^1^ Indeed, until a decade ago, it was widely assumed that we would see marked improvements only with gains in socioeconomic status and substantially strengthened health systems. However, the landmark work of Abhay Bang in the 1990s in a poor area of India with high newborn mortality demonstrated that simple services, delivered in the home by community health workers, could reduce neonatal mortality substantially.^2^ This prompted new interest in applying the kind of simple primary health care strategies that have been so effective in driving down mortality in older infants and children to the problem of newborn mortality.

## SEPSIS THROUGH CORD-STUMP SEEDING: CAN SOMETHING BE DONE?

Sepsis in the first week or two of life is a major cause of newborn deaths.^3^ People in many cultures apply substances to the freshly cut cord stump, such as ash, oil, butter, spice pastes, or mud. This and other unhygienic exposures to the fresh wound could well account for a significant proportion of newborn sepsis. Accordingly, cluster-randomized controlled trials have been conducted recently, first in Nepal^4^ and then in Bangladesh^5^ and Pakistan,^6^ to test **application of chlorhexidine—a commonly used antiseptic—to the umbilical cord stumps of newborns.** In the trials, chlorhexidine was applied daily to the cord stump for approximately a week (up to 14 days in the Pakistan study). In the Bangladesh study, in an additional treatment arm, chlorhexidine was applied only on the day of birth.

## HOW EFFECTIVE IS CHLORHEXIDINE?

At one time, chlorhexidine had been commonly used in newborn intensive care units in the West.^7^ But in 1999, the World Health Organization (WHO) stated that such antiseptic use was generally unnecessary (although WHO acknowledged it could still be suitable in settings with high risk of neonatal sepsis due to poor hygiene and called for more research in this area).^8^ Use of chlorhexidine for cord-stump care subsequently declined. However, Mullany's 2006 Nepal study showed a very promising result: among infants surviving long enough to enter the study (about 6 to 12 hours after delivery), mortality was about one-quarter lower among infants treated with chlorhexidine than in the comparison group.^4^ Moreover, mortality was *one-third* lower among those who received the first application within 24 hours of birth. (Among infants randomized to receive chlorhexidine but who got their first application *after day 1*, mortality was no different than among the comparison group.)

With these positive results, investigators began replication trials in Bangladesh,^5^ Pakistan,^6^ and later, in Tanzania^9^ and Zambia.^10^ The 2 recent South Asian trials were reported in the *Lancet* in early 2011.^5^^,^^6^ (Results from the 2 African trials will not be available for another year.) All 3 South Asian trials showed fairly similar, statistically significant protective effects against mortality (Table 1). In addition to chance, the differences in findings probably reflect some differences in environmental conditions and newborn care practices. The overall pooled effect size across the 3 studies was similar to the original Nepal trial.

**Table 1.** Neonatal Mortality Reduction From Chlorhexidine Cord Care

Country	% Reduction (intervention versus control)
Nepal^4^	24%
Pakistan^6^	38%
Bangladesh^5^	
Single application	20%
Multiple application	6% (NS)
**Pooled effect size**^11^	**23%**

Abbreviation: NS, not significant.

## WHAT IS THE POTENTIAL IMPACT ON NEONATAL MORTALITY?

All 3 trials completed to date have shown a protective effect, with a reduction in mortality risk of 23% in a pooled analysis.^11^ However, substantial neonatal mortality occurs very soon after birth, before chlorhexidine could be expected to have impact. Intrapartum (or asphyxia) deaths are estimated to account for 20% of global neonatal deaths.^3^ Thus, the remaining 80% of neonatal deaths—that is, non-asphyxia deaths, or those surviving beyond the first hours after birth—roughly correspond to the denominator population from which the chlorhexidine study subjects were drawn. So the expected contribution of this intervention in reducing the *overall* neonatal mortality rate could be estimated as 23% × 0.8, or about 18% (roughly 1 in 6 neonatal deaths averted).

An estimated 1 in 6 neonatal deaths could be averted with chlorhexidine cord care.

Is this a credible estimate of its effect? Skeptics have pointed out that in South Asia the *total* proportion of newborn deaths attributable to sepsis as principal cause is *only 14%*.^3^ However, chlorhexidine may contribute to reducing deaths otherwise attributed to complications of prematurity or tetanus, which, in South Asia, are estimated to account for 38% and 2% of newborn deaths, respectively.^3^ So, the observed effect size across the 3 studies appears reasonable.

## IS IT SUITABLE IN LOW-RESOURCE SETTINGS?

Chlorhexidine for umbilical cord care has many attractive features, notably:

- Addressing a problem with high population health burden
- Efficacy
- Low cost
- Simplicity
- Safety
- Acceptability
- Low regulatory requirements
- Health system compatibility
- Scalability
- Commercial viability

Beyond the issues of efficacy and expected impact on population health, important considerations in decisions to move chlorhexidine into routine use include cost, feasibility, simplicity, and acceptability.^12^

### Chlorhexidine Is Very Inexpensive

The principal costs are formulation, packaging, and transport, not the bulk cost of the raw ingredient. In Nepal, the bulk procurement cost of single-use chlorhexidine tubes is about US$0.23/newborn (personal communication with Prajwal Jung Pandey, Marketing Director of Lomus Pharm). In other settings, program managers may opt for a multi-day regimen, which would cost somewhat more—but would still be inexpensive in comparison with other routine labor and delivery interventions.

### Variety of Practical Distribution Channels Are Available for Chlorhexidine

When delivery takes place in a health facility, a health worker can apply chlorhexidine just after delivery. When deliveries happen at home, antenatal contacts or community health workers (a major channel in Nepal) can be used to distribute chlorhexidine late in pregnancy, or retail outlets can sell chlorhexidine—either linked to clean delivery kits or as a stand-alone socially marketed product—and the mother, another household member, or birth attendant can apply the product.

Manufacture of the chlorhexidine product is very straightforward and well-suited to local production in many country settings, resulting in lower costs and greater benefits to local economies.

**Figure:**
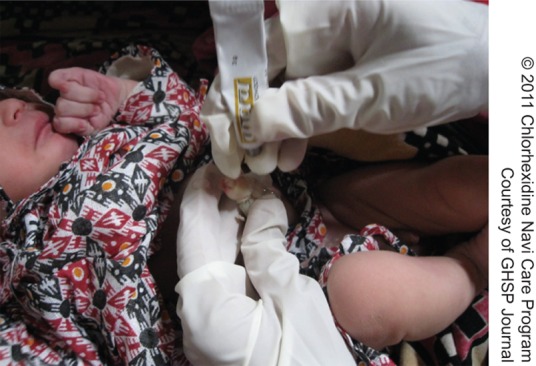
A health worker in Nepal applies chlorhexidine to a newborn.

### Simplified One-Time Regimen May Be Enough

It is clear from results of the Bangladesh and Nepal studies that, for effectiveness, chlorhexidine must be applied to the cord stump on the first day of life.^4^^,^^5^ While the 3 completed trials showed lower rates of visible cord infection in babies having multi-day application, it is unclear whether there is further mortality reduction from additional applications beyond day 1.

In Nepal, where the government is now moving forward with nationwide introduction of chlorhexidine, the Ministry has opted for a day 1-only regimen, based on the argument that this:

1. Minimizes messaging confusion (Advice to families will continue to be, “Keep it clean and dry, and don't put anything on it other than the chlorhexidine that was put on the first day.”)
2. Keeps costs down
3. Simplifies distribution

### What About Acceptability?

In formative work done in Bangladesh, Nepal, and elsewhere, household caregivers have consistently been very keen to use this product.^13^ It responds to a widespread perceived need, at the community level, for something to protect the cord from infection. Indeed, a *collateral benefit* demonstrated in the 3 completed trials was a marked reduction in frank infection of the cord stump.^4-6^

## ROLLOUT IN NEPAL

Nepal has had the benefit of conducting the first trial. Since then, the country has done formative acceptability and product development work,^13^ and then larger-scale piloting.^14^ A local manufacturer is producing a good-quality product, using a formulation conforming to household caregiver preferences. The Ministry of Health and Population, late in 2011, made a decision to proceed with nationwide implementation, and chlorhexidine is now being rapidly rolled out. Beyond modest external support for procurement during the first year of the program and for some ongoing monitoring and evaluation costs, the government of Nepal has committed to assuming the full expense of buying the commodity and other program costs from its own resources. As of July 2012, Nepal has scaled up this program to 26 of 75 districts, with distribution mainly through community health workers (as well as for among institutional deliveries), and is continuing rapid expansion. Neighboring Asian countries and several African countries are doing formative work and preparing to pilot. (See box on scaling up chlorhexidine for cord care, next page.)

The government of Nepal has assumed most costs to scaling up the chlorhexidine program nationwide.

**Box. Key Actions for Scaling up**

**Enlist Support from Policy Makers at Country Level**

- Engage, inform, and win over key *gatekeepers* and *opinion leaders* (for example, through early, one-on-one informational briefings and exchange of views with leaders in the pediatric community); foster and support *champions* who are well placed to influence opinion and decision-making; engage potential local *pharmaceutical* producers—early.
- Understand and work competently through local *policy and regulatory processes*, both *formal* (for example, registration with drug regulatory body, incorporation on Essential Medicines List) and *informal* (from the beginning, fully inform and elicit concerns from key government counterparts and opinion leaders).
- Ensure registration as an over-the-counter, not prescription, product.

**Sustain Program Momentum**

- Form a *technical working group* having Ministry of Health leadership and ongoing meaningful involvement by all key partners in directing the initiative (or incorporate mandate into an existing working group, if one already exists with suitable membership and mandate, for example, a Ministry-led newborn health or safe motherhood working group) and ensure effective functioning—on a sustained basis (with regular meetings, action points, follow-up).

**Appeal to Your Intended End-User**

- Conduct *formative research* to understand the potential user's current practices, perspectives, and preferences with respect to appropriate care of the newborn cord stump.
- *Start where the user is now*, “bridging from the known to the new,”^15^ for example using formulation and packaging that resemble current products used for cord application.

**Use Simple Approaches and Messages**

- For example, day-of-birth-only application, if appropriate.

**Tailor Delivery Strategy to Existing Channels**

- Use *antenatal care* (ANC) if current ANC attendance rates are fairly high.
- Use *community health workers* if they are already reaching a large proportion of pregnant women.
- Enlist *private providers and NGOs* as appropriate.
- Add chlorhexidine to clean delivery-kit programs, if they are already reaching large numbers.

**Plan for Ultimate Scale and High Coverage**

- Product: Secure long-term arrangements for *procurement* of quality product and ensure adequately robust *supply chain*.
- Providers: Address provider skills, attitudes, and behavior for ultimate large scale.

**Phase Scale Up**

- Start with a *learning phase*, implementing at limited scale (for example, within one district) but under conditions closely approximating what you would expect when institutionalized and running as a normal program; *rigorously monitor* during this phase, and then, based on what has been learned, revise and streamline the approach for at-scale implementation.

**Monitor Actively**

- Through all phases, from early learning to at-scale implementation, ensure continued sound performance management—at all levels, *monitoring* important aspects of program performance (notably coverage) and actively addressing identified performance issues. This is likely to entail incorporating chlorhexidine coverage into the *routine health information system*, and taking measures to ensure that coverage is being monitored at all levels, as a basis for taking action to ensure good performance.

## SUPPORTIVE GLOBAL RECOMMENDATIONS MAY BE IMMINENT

Given the new evidence that has recently accrued, in September 2012 the WHO convened an expert consultative meeting, which recommended incorporating chlorhexidine into program use. Having received this expert input, WHO is now completing its own internal review process and is expected to release a statement soon on chlorhexidine use for cord care.

In September 2012, a WHO expert consultative group recommended chlorhexidine for cord care.

## DOES CHLORHEXIDINE DESERVE THE BILLING OF GAME-CHANGER?

Consider the following:

1. **Huge potential impact**. Our best estimate of efficacy is that its use (at least in a South Asian setting) has the potential to reduce overall newborn mortality risk by up to 18%. Given an estimated 3.1 million newborn deaths per year,^1^ an 18% decline would represent over half a million fewer deaths per year globally.
2. **Safe and stable**. For this use, it has effectively no toxicity risks or potential for misuse,^16^ and no special storage requirements.^7^
3. **Remarkably inexpensive and cost effective**. The commodity cost per newborn is about US$0.23 (in Nepal, where a first-day-only regimen is used). With a neonatal mortality rate of 30 per 1,000 live births (typical of South Asia)^17^ and a number-needed-to-treat of 185 per averted death, this translates to a commodity procurement cost of about US$45 per averted death. By piggybacking on other program efforts, rollout and ongoing program costs should be low.
4. **Highly acceptable and functional**. As a **behavior change** challenge, in many settings we would merely be applying a scratch to a pre-existing itch. To date, consumer acceptability studies have found a near universal desire to use the product.^13^ Furthermore, we have not seen problems with correct use in pilot studies.^14^
5. **Multiple possible distribution channels and potential for direct consumer use.** Even in settings where deficiencies in the health care system preclude delivery of most other efficacious newborn interventions at high coverage, distribution and utilization of chlorhexidine does not face the same constraints; a health worker is not required and multiple channels can be used for distribution.

Preventing 1 in 6 neonatal deaths would be huge. “Game-changer” seems, to us, a pretty good fit.
